# Impact of an innovative bundled payment to TB health care providers in China: an economic simulation analysis

**DOI:** 10.1186/s12913-024-11034-8

**Published:** 2024-05-03

**Authors:** Pengyu Xu, Yazhen Ying, Debin Xu, Shitong Huan, Lindu Zhao, Hong Wang

**Affiliations:** 1 School of Economics & Management, Southeast University, No. 2, Sipailou, Xuanwu District, Nanjing, Jiangsu Province 210096 China; 2 National Institute of Healthcare Security Capital Medical University, Beijing, China; 3 Bill & Melinda Gates Foundation, Seattle, WA USA

**Keywords:** Health policy, Tuberculosis, Descriptive study, Health economics, Control strategies

## Abstract

**Background:**

Tuberculosis is the second most deadly infectious disease after COVID-19 and the 13th leading cause of death worldwide. Among the 30 countries with a high burden of TB, China ranks third in the estimated number of TB cases. China is in the top four of 75 countries with a deficit in funding for TB strategic plans. To reduce costs and improve the effectiveness of TB treatment in China, the NHSA developed an innovative BP method. This study aimed to simulate the effects of this payment approach on different stakeholders, reduce the economic burden on TB patients, improve the quality of medical services, facilitate policy optimization, and offer a model for health care payment reforms that can be referenced by other regions throughout the world.

**Methods:**

We developed a simulation model based on a decision tree analysis to project the expected effects of the payment method on the potential financial impacts on different stakeholders. Our analysis mainly focused on comparing changes in health care costs before and after receiving BPs for TB patients with Medicare in the pilot areas. The data that were used for the analysis included the TB service claim records for 2019–2021 from the health insurance agency, TB prevalence data from the local Centre for Disease Control, and health care facilities’ revenue and expenditure data from the Statistic Yearbook. A Monte Carlo randomized simulation model was used to estimate the results.

**Results:**

After adopting the innovative BP method, for each TB patient per year, the total annual expenditure was estimated to decrease from $2,523.28 to $2,088.89, which is a reduction of $434.39 (17.22%). The TB patient out-of-pocket expenditure was expected to decrease from $1,249.02 to $1,034.00, which is a reduction of $215.02 (17.22%). The health care provider’s revenue decreased from $2,523.28 to $2,308.26, but the health care provider/institution’s revenue-expenditure ratio increased from -6.09% to 9.50%.

**Conclusions:**

This study highlights the potential of BPs to improve medical outcomes and control the costs associated with TB treatment. It demonstrates its feasibility and advantages in enhancing the coordination and sustainability of medical services, thus offering valuable insights for global health care payment reform.

## Background

According to the latest WHO 2022 Global Tuberculosis (TB) Report, TB is the second most deadly infectious disease after novel coronavirus pneumonia (COVID-19) and the 13th leading cause of death worldwide. There were 10.6 million TB infections, which is equivalent to 134 cases per 100,000 people, and nearly 1.6 million deaths from TB. The number of confirmed cases of TB reached 6.4 million globally in 2021, which increased from 5.8 million in 2020. Among the 30 countries with a high burden of TB, China ranks third in the estimated number of TB cases after India and Indonesia. In 2021, 75 of 136 low- and middle-income countries reported of needing more funding to fully implement their national TB strategic plans. The total reported funding gap is $1.6 billion, with the largest gaps observed in Indonesia ($340 million), Nigeria ($256 million), the Philippines ($164 million), and China ($109 million) [1]. Although the Chinese government has provided free services for basic TB examinations and treatment, as well as the fact that health insurance policies also cover essential services/medicines within a defined insurance benefits package, TB patients still experience a considerable financial burden [2, 3]. According to the 2022 WHO data, among the top four countries with the largest funding gaps mentioned above, the largest average expenditure per TB patient is in China ($848.078), followed by Nigeria ($693.941), Indonesia ($241.342), and the Philippines ($190.077).

A previous study demonstrated that TB service costs and patients’ financial burdens are high, which is primarily due to the high use of inpatient services [4]. Another study from Zhenjiang, China, also demonstrated that despite advances in TB insurance policies, there are still significant costs associated with TB diagnosis and treatment; moreover, due to the high hospitalization rate, TB patients still experience a considerable financial burden [5]. These studies call attention to developing a more comprehensive benefits package for the TB service that is included in the health insurance scheme, improving the utilization of TB treatment guidelines, and reforming health insurance regulations for TB patients to receive appropriate health care services.

Case-based Payment (CBP) is a payment mechanism that has been widely adopted throughout the world. China introduced this method in 2010 to replace the traditional fee-for-service (FFS) payment method. There are currently two approaches to CBP.

The first approach involves grouping cases based on the diagnosis and severity of the condition. Initially, cases are categorized by the diagnosed disease. These primary groups are then subdivided into three severity-based subgroups: light (Group A), medium (Group B), and serious (Group C). Group A typically includes uncomplicated cases of a disease, while Group B covers cases with common complications. Group C, reserved for severe complications, involves a smaller patient cohort. Standard clinical pathways are established for Groups A and B with fixed payment standards set by health insurance organizations, which pay according to these predetermined rates. Conversely, Group C still operates under the fee-for-service model due to the variability and complexity of the cases.

The second method segments payments based on the cost associated with different case subgroups. This is done by categorizing diseases into three broad categories—surgical, nonsurgical, and pediatric treatments—based on the operation codes from the International Classification of Diseases, Ninth and Tenth Revisions (ICD-9 and ICD-10). Patients are then grouped according to their specific diagnoses into these categories. Each category is further divided into three cost-based segments: high, medium, and low. Payment standards are then assigned to each segment within these groups, and health insurance organizations settle accounts with medical institutions based on the proportion of cases in each segment and the corresponding payment standard [6–8].

Currently, health insurance departments in China mostly adopt CBPs for TB patients. China classifies various types of TB, including pulmonary, extrapulmonary, drug-sensitive and drug-resistant cases. The principle of CBP is the same for all types, although the specific treatment plans may vary slightly. The management expenses for all TB patients in China are primarily covered by the National Health Security Administration (NHSA). Moreover, China also has several national TB prevention and control programs, which are under the jurisdiction of the NHSA. China’s civil affairs relief departments also provide extra financial assistance for patients in particularly difficult economic situations; however, this is separate from the NHSA [8].

Over the last 13 years (from 2010 to the present), the implementation of a CBP system for TB has significantly improved the rate of initial diagnosis, the detection rate of patients, and treatment compliance, as well as reduced the loss of cases and promoted the standardized treatment and management of TB patients [9–11]. Compared to FFS, CBPs can reduce high medical costs and control the rapid growth of medical expenses [11]. Additionally, through SWOT analysis of interviews with stakeholders and analysis of routine hospital data, scholars have found that CBPs for TB services can alleviate the economic burden on patients, standardize doctors’ diagnostic and treatment behaviors, control medical costs, and ensure the quality of services [12]. However, there are certain shortcomings and problems with CBPs. CBPs are usually made on the basis of a specific case or disease, which may lead to fragmentation of the medical process into different stages or parts, which correspondingly may lead to a decline in the coordination and continuity of medical services, as different providers may focus only on their own scope of payment to neglect the overall treatment outcome. CBP emphasizes payment on a case-by-case basis, which may only be able to comprehensively cover some patients’ medical needs and treatment processes. Due to the fact that CBPs are paid based on specific cases or diseases, providers or doctors may provide more medical services to increase their income. This may lead to overtreatment and unnecessary medical manipulation, thus increasing the cost of care with no apparent benefit. In particular, a considerable financial burden remains for most TB patients, especially poor patients who are hospitalized [13].

In this context, NHSA has developed an innovative payment mechanism (the bundled payment, BP) and implemented it in the nationwide provider payment pilots performed by the NHSA. In this payment model, health care insurance institutions or payers do not pay for individual service items but make lump-sum payments based on the total cost of a series of related medical services. These services may involve a specific medical procedure, treatment process, or treatment cycle for a disease. According to the literature, U.S. Medicare has been piloting BP programs since the late 1980s [14]. One example is the pilot organized by the Center for Medicare and Medicaid Innovation (CMMI), in which a BP is developed for the Coronary Artery Bypass Grafting (CABG) and cardiac care. The results showed that seven participating providers saved $42.3 million (approximately 10% of expected expenditures) over five years [15]. In 2007, a BP approach was introduced as a critical intervention in the Netherlands’ comprehensive diabetes-focused care program. The results showed that this payment program achieved risk sharing, increased the frequency of care provided by providers, decreased blood pressure and cholesterol levels, and significantly improved the health status of patients with diabetes [16]. In 2010, the Obama bill reformed Medicare’s payment system and officially introduced BPs on a large scale. When regarding implementation effects, BPs have promoted collaboration among different providers and effectively increased the sense of risk sharing among providers [17].

The following factors are some of the main differences observed between CBPs and BPs. Firstly, CBPs are payments according to cases or diseases, whereby the medical institution or doctor receives a fixed sum based on the patient’s diagnosis or treatment. BPs bundle a series of related health care services to pay a fixed fee for the entire process or phase. Secondly, CBPs typically focus on treating a single case or disease, with payment based on a specific diagnostic code or disease classification. BPs encompass a much broader scope and can include a range of related health care services, such as all of the costs associated with a surgical procedure (including surgery, anesthesia, hospitalization and others). Thirdly, CBPs are usually one-time, such as a single payment for each patient or disease. BPs involve a much longer time period and can encompass the entire process ranging from admission to discharge or a specific stage of care. Furthermore, in CBPs, the provider or doctor usually charges a fixed fee, which covers the entire treatment of the case or disease. In BPs, the allocation of fees may be adjusted based on the relative value of different medical services or resource utilization. Lastly, CBPs may incentivize physicians to provide comprehensive and high-quality care because their income is tied to the success of their treatment. BPs can incentivize providers or physicians to deliver efficient and coordinated care at specific stages or processes to ensure quality and effectiveness [17–22].

The objectives of this study were to simulate the effects of this BP approach on different stakeholders (including TB patients, payer/health insurance agencies, and TB health care providers) within the health care system to provide analytical evidence to support the adoption of these newly developed health care provider payment methods in overall health care provider payment interventions. Moreover, this study aimed to utilize such an effective policy tool to lower the economic burden on TB patients, decrease their likelihood of hospitalization, increase their chances of seeking outpatient care, and alleviate their suffering. Second, it seeks to improve the financial stability of health care providers, assist the government in optimizing related policies, and reduce the financial risks to health insurance funds. Finally, we hope to provide some experiences or models that other countries or regions can reference.

We used Monte Carlo simulation for our study. According to the literature, the use of simulation methods to examine the ex-ante impact of health policy changes has been able to help researchers in gaining insights into which variables are most important to what is being evaluated, thus allowing us to explore possible problems and scenarios without the need for real experiments. For example, Thurecht et al. [23] predicted the number of Australians aged 25 years and older who would be expected to have prediabetes and type 2 diabetes over a 45-year simulation period by simulating the development of a diabetes model ex ante. The authors simulated disease control in terms of variables such as blood glucose levels, cholesterol levels, body weight and blood pressure. The model produced a wide range of epidemiological and economic outputs to assess the current and projected impact of disease on people. The number and cost of complications associated with type 2 diabetes and the impact on the level of diabetes control were also successfully predicted [24]. Lin et al. [25] considered simulation methods as a tool to estimate the health impact of changes in risk factor prevalence in a population, and the results can be directly used in health policy development to set targets or quantify different scenarios of future changes in risk factor prevalence; alternatively, the results can be used as input to formal decision-making processes, such as cost-effectiveness studies. Freebairn et al. [26] argued that simulation methods can also synthesize and leverage existing evidence, data, and expert local knowledge to examine the likely impact of alternative policy and service delivery options in a robust, low-risk, and low-cost manner. For example, Freebairn et al. [27] combined senior public health policy-makers and health service providers to provide experience and successfully conducted simulation modelling of three health policy case studies (alcohol-related harm, childhood obesity prevention, and diabetes in pregnancy).

The most significant contribution of our research is the in-depth analysis and evaluation of the BP mechanism, thus demonstrating that the BP approach developed by the NHSA is viable and beneficial for TB patients in China, which provides an innovative perspective. This new payment model could significantly improve medical service quality, reduce patients’ financial burden, and enhance the financial stability of health insurance funds, especially in TB treatment. By analysing the impact of case-based and BPs on the behaviour of medical service providers and the economic burden on patients, we emphasized the importance of improving payment mechanisms to enhance the quality of medical services and patient satisfaction. Furthermore, we demonstrated that BPs could help to improve the coordination and continuity of medical services, which is particularly important for managing chronic and complex diseases. By fostering collaboration among multidisciplinary teams, BPs can improve medical services throughout the disease treatment cycle. Ultimately, our research aims to extend such a payment method from pilot projects focused on a single disease to other diseases (especially those such as chronic diseases that require emphasis on prevention) and implement it in all of the regions across the country.

Compared to previous studies on different health care payment methods, our research differs in the following ways. Firstly, past studies have focused on evaluating a single payment method (such as FFS or CBP), whereas our research introduces the innovative mechanism of BPs, thus providing a multidimensional analysis of medical payment methods. It analyses the effects of CBPs and specifically explores how BPs impact cost control, the behaviour of medical service providers, and patients’ financial burden in TB treatment. In addition, our research not only focuses on the direct impact of payment methods on medical costs and service quality (which is typically the only consideration) but also considers how reforming medical payment mechanisms can improve the financial stability of medical service providers, assist the government in optimizing related policies, and reduce the financial risks to health insurance funds. These recommendations provide strategic support for the comprehensive reform of the medical system. Furthermore, unlike studies that are usually limited to a single country or region, our research offers an international and regional perspective by comparing the implementation experiences of BPs in China and other countries (such as the USA and the Netherlands). This helps in understanding the feasibility and effects of BP strategies in different medical systems. Lastly, by analysing the economic burden of TB patients, our research delves into the impact of medical payment methods on the economic situation of specific patient groups. This analysis emphasizes the importance of considering patients’ economic affordability when designing medical payment policies.

## Methods

### Bundled payment design

This pilot used the BP concept to design a TB provider payment intervention. A BP is a payment method that is used to calculate the sum of all of the clinically defined actions of health care delivery. The advantage is that the health care service provider and payer share financial risks. If the cost of care is less than the BP amount, the provider retains the difference. However, if the cost exceeds the amount paid, the provider bears the loss [18, 19, 28].

The key features of the design of this innovative payment intervention in this pilot include the following.

#### Identification of TB patients

The TB cases included primary and secondary TB diagnoses with the ICD-10 codes A15.0, A15.1, A15.2, A15.3, A15.4, A15.7, A16.0, A16.1, A16.2, A16.3, and A16.7.

#### Bundled payment rate estimation

The payment rate covers outpatient and inpatient services per patient per year with a defined targeted hospitalization rate. The payment rate is estimated by using previous 3-year expenditure data (2019–2021) of the infectious disease hospitals in this pilot city.

#### Insurance payment

The total annual amount of health insurance payments to health care providers/institutes is based on the estimated number of TB patients multiplied by the BP price and the insurance reimbursement rate. The BP price is individualized and determined based on a uniform TB treatment plan that is established nationwide, taking into account the economic differences in prices in each province/region. The reimbursement rate is based on the previous 3-year rates (2019–2021).

#### Monitoring and evaluation

As part of the intervention, the pilot project introduced a series of monitoring and evaluation activities to reinforce the implementation of the interventions with the following key areas of focus: (1) the intervention has been implemented according to the design, (2) the standardized treatment has been used appropriately, (3) the incentive, the potential savings that are kept or potential loss borne by the providers has been put into effect, and (4) the monitoring and supervision activities have been performed appropriately.

The pilots of this BP intervention for TB services were performed in four cities, with approximately 1.98 million people covered by the Urban Employee Basic Medical Insurance (UEBMI) and the Urban Resident Basic Medical Insurance (URBMI). Given that BP is a flat rate per patient per year, it is expected that health care providers will strive to reduce service costs, especially regarding unnecessary hospitalization services. Therefore, they can generate more savings to make their financial status more sustainable. These changes will also likely reduce TB patients’ financial burden and the financial risks of health insurance.

### Analytical model, variables, and data sources

We established our analytical model through the following three steps: before the intervention, after the intervention, and after the intervention with the uncertainties during the implementation. All of the calculations below indicate the amount per TB person by year.

We defined ‘medical expenditure’ as ‘E(T)’ in the analysis, which is the key outcome variable. Without the intervention, medical expenditures consisted of two components: outpatient and inpatient expenditures. Therefore, we defined ‘the outpatient medical expenditure’ as ‘E(O)’ and defined ‘the inpatient medical expenditure’ as ‘E(I)’. This can be expressed with Eq. (1) below, where i represents each patient.

1 $${E\left(T\right)}_{i}={E\left(O\right)}_{i}+{E\left(I\right)}_{i}$$

With the intervention, we expected that due to the financial incentive introduced by the BP and patient treatment guideline application, a certain percentage of patients would shift from inpatient to outpatient care, with a decrease in the number of inpatients and a corresponding increase in the number of outpatients.

This also indicates a decrease in inpatient expenditures and a corresponding increase in outpatient expenditures. Before the intervention, we defined ‘the outpatient medical expenditure’ as ‘*O*_*0*_’ and ‘the inpatient medical expenditure’ as ‘*I*_*0*_’. After the intervention, $\Delta{E\left(O\right)}_{i}$ represents the increase in outpatient medical expenses. $\Delta{E\left(I\right)}_{i}$ represents the decrease in expenditures on inpatient medical services. $\Delta{R\left(O\right)}_{i}$ represents the amount of change in the outpatient rate, and $\Delta{R\left(I\right)}_{i}$ represents the amount of change in the hospitalization rate. $\Delta{R\left(O\right)}_{i}$ and $\Delta{R\left(I\right)}_{i}$ are two negatively correlated indicators, so $\Delta{R\left(O\right)}_{i}$ is equal to $\Delta{R\left(I\right)}_{i}$. ‘*N’* represents the number of TB patients. $N\times \Delta{R\left(O\right)}_{i}$ represents the increase in the number of outpatients, which corresponds to the decrease in the number of inpatients ($N\times \Delta{R\left(I\right)}_{i})$. ${O}_{0}\times N\times \Delta{R\left(I\right)}_{i}$ represents the total increase in outpatient expenditure resulting from the increase in the number of outpatients. $\frac{{O}_{0}\times N\times \Delta{R\left(I\right)}_{i}}{N}$ represents the increase in outpatient expenditure per TB patient. ${I}_{0}\times N\times \Delta{R\left(I\right)}_{i}$ represents the total decrease in inpatient expenditure resulting from the decrease in the number of inpatients. $\frac{{I}_{0}\times N\times \Delta{R\left(I\right)}_{i}}{N}$ represents the decrease in inpatient expenditure per TB patient. After sorting and simplifying, the estimation of the medical expenditure per TB patient by year can be expressed in Eq. (2) below.

2 $${E\left(T\right)}_{i}={E\left(O\right)}_{i}+{E\left(I\right)}_{i}={(O}_{0}+\Delta{E\left(O\right)}_{i})+({I}_{0}-\Delta{E\left(I\right)}_{i})={(O}_{0}+\frac{{O}_{0}\times {\text{N}}\times \Delta{R\left(O\right)}_{i}}{{\text{N}}})+({I}_{0}-\frac{{I}_{0}\times {\text{N}}\times \Delta{R\left(I\right)}_{i}}{{\text{N}}})={(O}_{0}+\frac{{O}_{0}\times {\text{N}}\times \Delta{R\left(I\right)}_{i}}{{\text{N}}})+({I}_{0}-\frac{{I}_{0}\times {\text{N}}\times \Delta{R\left(I\right)}_{i}}{{\text{N}}}) =\left[{O}_{0}\times \left(100\%+\Delta{R\left(I\right)}_{i}\right)\right]+[{I}_{0}\times \left(100\%-\Delta{R\left(I\right)}_{i}\right)]$$

**Fig. 1:**
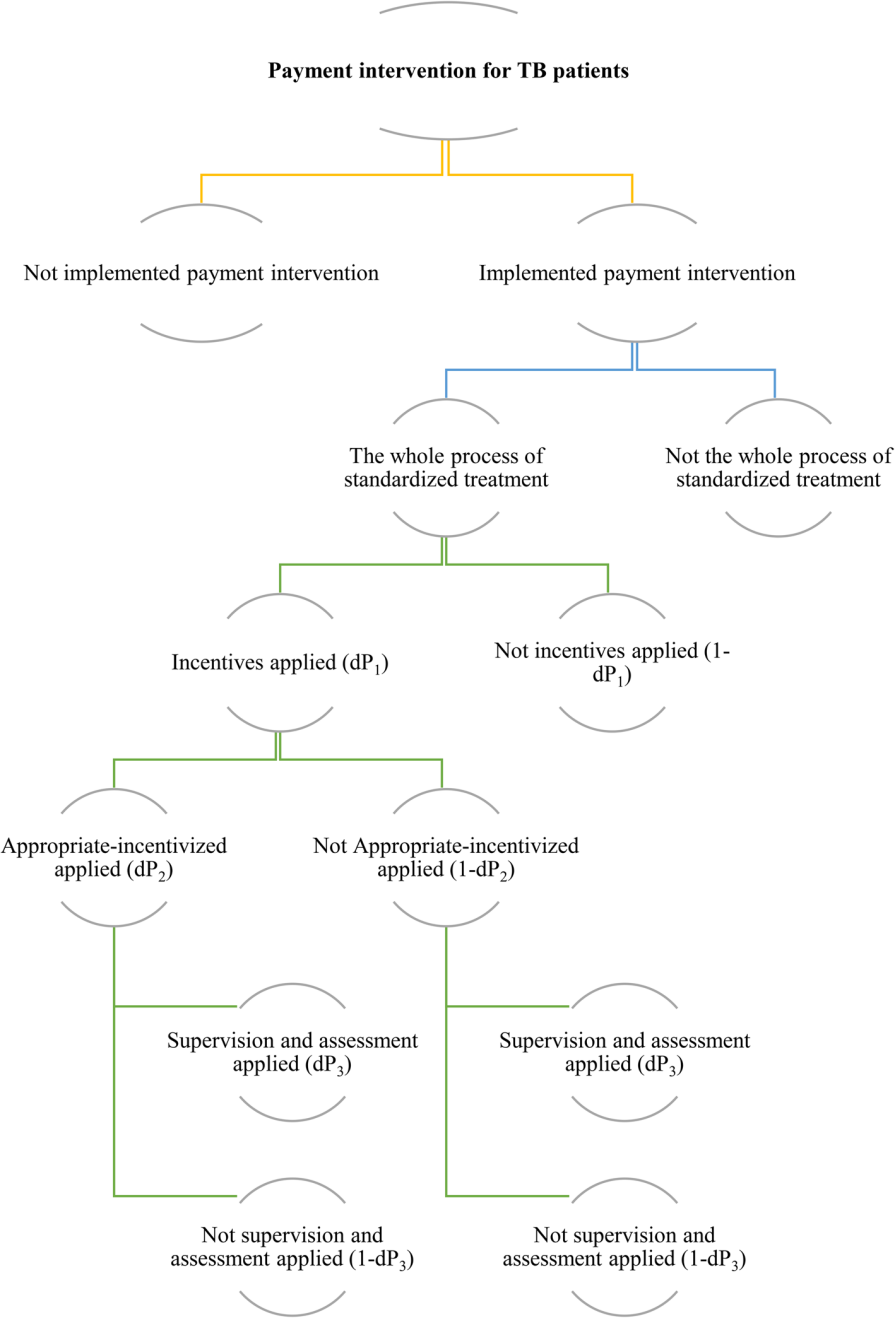
Diagram of the decision tree

As we assumed, the implementation of this intervention will encounter some uncertainties, ranging from whether the payment intervention is implemented or not in the pilot areas, whether the standardized treatment guideline has been followed appropriately or not, whether incentives are applied or not, whether appropriate-incentivized factors are applied or not, and whether supervision and assessment are applied or not. All of these uncertainties will affect the intervention results, thus decreasing the effects of reducing the hospitalization rate and controlling TB service expenditures. Supervision and assessment can influence the effectiveness of incentive implementation and thereby the implementation of standardized treatment. We used a decision tree to map the alternative outcomes associated with these uncertainties, as shown in Fig. 1.

We subsequently included these uncertainties in estimation Eq. (3) below.

3 $${E\left(T\right)}_{i}={\{O}_{0}\times \left[1+\left(100\%-R\left(I\right)\right)\times {dP}_{1}\times {dP}_{2}\times {dP}_{3}\right]\}+{\{I}_{0}\times [1-(100\%-R(I))\times {dP}_{1}\times {dP}_{2}\times {dP}_{3}]\}$$

We introduced the targeted hospitalization rate *R(I)* in Eq. (3) to represent the “ideal” hospitalization rate under the intervention. We assumed that the original hospitalization rate was 100%, which is the worst-case scenario. $\Delta{R\left(I\right)}_i\;\mathrm{is}\;\mathrm{equal}\;\mathrm{to}\;\left(100\%-R\left(I\right)\right).$ This target will be affected by the uncertainties of (1) the probability of whether incentives are applied or not, which is represented by dP_1_; (2) the probability of whether appropriate incentives are applied or not, which is represented by dP_2_; (3) the probability of whether supervision and assessment are applied or not, which is represented by dP_3_; and (4) the values of dP_1_/dP_2_/dP_3_ are all assumed to be in the range of (0–100%) for the simulation analysis.

The data that were used for this analysis are taken from five main sources in one of four pilot cities. The first data source is the claim records of TB services from the health insurance agency from 2019 to 2021. These data have been used for TB patient service utilization analysis. The second data source is the local Centre for Disease Control (CDC). These data have been used to estimate the TB hospitalization rate and annual number of TB patients. The third source of data is the local statistics yearbooks. These data have been used to estimate the revenue and expenditure of health care facilities that provide services to TB patients. The fourth dataset comes from the China Technical Guidelines for TB Prevention and Control (2020 Edition) and the Local Medical Service Price Catalogue. The fifth figure is from the National Statistical Office. These two sets of data are used to calculate the standard cost of the service package for general TB patients (as shown in Table 1).

**Table 1:** List of data sources

Characteristics	Variables	Source of data
Outpatient medical expenditure	O_0_	The claim records of TB services from the health insurance agency from 2019 to 2021
Inpatient medical expenditure	I_0_	
Actual reimbursement ratio (%)	-	
Number of TB patients	N	The local Centre for Disease Control (CDC)
Target hospitalization rate	R(I)	
Revenue for TB health care providers/institutes	-	The local statistics yearbook
Expenditure for TB health care providers/institutes	-	
Standard cost of service package for general TB patients	-	Technical Guidelines for TB Prevention and Control in China (2020 edition) & Local Medical Service Price Catalogue
Consumer price index for medical services	CPIMS	National Statistical of Statistics

### Statistical analysis

A Monte Carlo randomized simulation model was applied to the data analysis. Clark first introduced the Monte Carlo method in 1961 [29]. Most scholars apply it to computational simulations [30]. Richter and Mauskopf [31] applied the Monte Carlo simulation model in the health care domain and used it to economically evaluate health care interventions. A Monte Carlo simulation experiment was used to evaluate the cost-effectiveness of FFS and BP for breast cancer patients [32]. Arenas et al. [33] used Monte Carlo simulations to estimate the health impact of common preventive health interventions applied to individuals in quality-adjusted life-years (QALYs). In this experiment, 100 Monte Carlo simulations were used to assess the economic impacts of changes in policy. Using existing evidence and data, we can examine the possible impacts of the implementation of BP policies in a robust, low-risk, and low-cost manner.

## Results

### The estimated input values and assumptions for the simulation analysis and other data are used

The inputs include *O*_*0*_ (outpatient medical expenditure), *I*_*0*_ (patient medical expenditure), and *R(I)* (target hospitalization rate). Parameters to be used in calculating the results include the actual reimbursement rate (%), the number of TB patients, the income of TB providers/institutions, the expenditure of TB providers/institutions, the standardized cost of a package of services for a general population of TB patients, and the Consumer Price Index for Health Services (CPIHS) (shown in Table 2).

**Table 2:** The input values and assumptions for this simulation analysis

Characteristics	Variables	Values and assumptions
Outpatient medical expenditure	*O*_*0*_	$660.44
Inpatient medical expenditure	*I*_*0*_	$1,862.84
Target hospitalization rate	*R(I)*	30% or 50%
Actual reimbursement ratio (%)	-	50.50%
Number of TB patients	N	4,920
Revenue for TB health care providers/institutes		$17,550,000.00
Expenditure for TB health care providers/institutes		$18,610,000.00
Standard cost of service package for general TB patients	-	$1,225
Consumer price index for medical services	CPIMS	102.3

Below is specific information and an explanation of the input values and assumptions that were used for the analysis and some of the data that were used in the calculation of the results.

#### The estimation of total annual expenditures per TB patient without intervention

We used an exchange rate ranging from ¥6.45 to $1.00 (the average exchange rate in 2021) to convert expenditures/costs to USD. We applied the ratio of 1:2:7 weights to the data from 2019–2021 to predict the values of *O*_*0*_ (outpatient medical expenditure), *I*_*0*_ (patient medical expenditure), *N* (number of TB patients) and the *actual reimbursement ratio* (%) (as shown in Table 3). The 1:2:7 ratio comes from the DIP (big data diagnosis-intervention package) program in China. The Chinese government began piloting this big data-based payment by disease fraction in 2020 as a supplement to and extension of DRGs (diagnosis-related groups). The program calculates the average hospitalization cost to comprehensively reflect the development trend of disease costs over the years by using the past data of the last three years in a time-weighted form to calculate the average value of that cost. For example, for the current year of 2022, we use the historical data of the previous three years, according to the ratio of 2019:2020:2021 = 1:2:7.

**Table 3:** Indicators for TB patients 2019–2022

				Unit: $
**Variables**	**2019**	**2020**	**2021**	**2022 (1:2:7)**
*O*_*0*_	445.05	598.62	708.88	660.44
*I*_*0*_	1,811.66	1,676.29	1,923.45	1,862.84
*N*	5,745	5,501	4,636	4,920
*Actual reimbursement ratio* (%)	53.56	51.79	49.69	50.50

#### Target hospitalization rate (R(I))

The targeted hospitalization rate (*R(I)*) is considered an “ideal” hospitalization rate that the project is aiming to achieve over time. A hospitalization rate of 30%, which was introduced by the China National Health and Family Planning Commission/China CDC in its innovative TB control and prevention program in four Chinese cities [34], was used as the targeted hospitalization rate in this simulation analysis. In addition, we used *R(I)* = 50% for the sensitivity analysis, given the potential challenges of reducing the hospitalization rate significantly in the short term.

#### The estimation of the revenue and expenditure of health care providers/institutes without the intervention

According to the Infectious Disease Hospital data related to the pilot city in the 2018–2020 health sector financial accounts report, we use 1:2:7 to predict the potential revenue, expenditure, and surplus yield from TB services provided by health care providers in 2021 and 2022 (Table 4). The results showed that health care providers/institutions would experience a financial loss due to the fact that the revenue is lower than the expenditure.

**Table 4:** Revenue and expenditure for TB health care providers/institutes in the pilot city, 2018–2022

					Unit: million $
**Items**	**2018**	**2019**	**2020**	**2021 (1:2:7)**	**2022 (1:2:7)**
Revenue	19.50	22.40	15.66	17.39	17.55
Expenditure	20.39	22.38	17.12	18.50	18.61
Surplus (Difference between revenue & expenditure)	-0.89	0.02	-1.46	-1.11	-1.07
Surplus/Revenue ratio	-4.57%	0.07%	-9.34%	-6.38%	-6.09%

#### The estimation of standardized TB patient treatment costs

For comparison purposes, we also estimated the standardized TB patient treatment cost based on services listed in the Technical Guidelines for TB Prevention and Control in China (2020 edition) [35]. The probabilities of occurrence of the services and the price of each treatment item and medicine were determined according to the local medical service price catalogue. The estimation of standardized treatment costs per patient $=\sum {N}_{i}\times {P}_{i}\times {\pi }_{i}$, where *N* represents the number of service items, *P* represents the price of the service items, *π* represents the probability of the occurrence of the services, and *i* represents a specific service item. The results of the standard cost measurement are shown in Table 5.

**Table 5:** Standard cost of service packages for general TB patients

	Unit: $
**Medical treatment items**	**General type TB**
Anti-tuberculosis drugs	73.64
Outpatient check-ups examinations	328.06
Inpatient check-ups	472.09
Adverse reaction management	120.00
Complication management	151.94
Other, e.g., molecular biology diagnostics	24.81
**Total**	**1,170.54**

According to the abovementioned data, the total cost of the whole process of standardized treatment for TB patients was $1,170.54 in 2020. This value will certainly change to some extent in 2022 due to factors such as economic levels and spending power. We project this change based on the 2020 consumer price index for medical services (CPIMS) published by the National Bureau of Statistics in China. The CPIMS was 102.3 in 2020, and we predicted that the whole process of standardized treatment for TB patients will be $1,225 in 2022.

### Results of Monte Carlo randomized simulation experiments for general TB patients

We used Eq. (3) as our simulation model to predict the total annual expenditure per TB patient. We assume that treatment effects remain consistent across payment mechanisms. Monte Carlo randomized simulation experiments were conducted 100 times with the relevant inputs described above. The estimated results showed that, if R(I) = 30%, the average value after the intervention from the experimental results is $2,088.89, the minimum value is $1,695.91, and the maximum value is $2,449.82. If R(I) = 50%, the average value after the intervention from the experimental results is $2,243.67, the minimum value is $1,925.55, and the maximum value is $2,451.77 (Table 6). These estimated results are above the value of $1,225, which represents the estimated standardized treatment costs, given the uncertainties of the implementation of the intervention. The estimated results also showed that after the intervention, the total annual medical expenditure per TB patient was lower than the total annual medical cost before the intervention, which was approximately $2,523.28 per patient per year.

**Table 6:** Total annual medical expenditures per patient before and after the intervention in 2022

			Unit: $		
**R(I)**	**Standardized treatment**	**Before intervention**	**After intervention**		
			**Average value**	**Minimum value**	**Maximum value**
30%	1,225.00	2,523.28	2,088.89	1,695.91	2,449.82
50%			2,243.67	1,925.55	2,451.77

### The estimated financial “benefits” of each stakeholder in the system

The total annual expenditure per TB patient is mainly composed of health insurance payments (HIPs) and out-of-pocket (OOP) payments. The results in Table 7 show that without the intervention, the total annual expenditure per TB patient is expected to be approximately $2,523.28. The OOP expenditure is $1,249.02, and the HIP expenditure is $1,274.26 per patient, with a reimbursement rate equal to 50.50% (Table 3 by 2022).

**Table 7:** Changes in revenue and expenditure per patient before and after the newly developed payment

								Unit: $
	***Before* intervention value**	***After* intervention value -**R(I)** = 30%**			***After* intervention value -**R(I)** = 50%**			**Description** (**E:** Expenditure; **R:** Revenue; **AR:** Additional Revenue; **ESE:** Expected savings expenditure)
(Per TB patient by year)	Average	Average	Minimum	Maximum	Average	Minimum	Maximum	
Medical (M) expenditure	2,523.28	2,088.89	1,695.91	2,449.82	2,243.67	1,925.55	2,451.77	E(M)
Out-of-pocket (OOP) expenditure	1,249.02	1,034.00	839.48	1,212.66	1,110.62	953.15	1,213.63	E(OOP) = E(M) × 49.5%
Health Insurance Payments (HIP) expenditure	1,274.26	1,274.26	1,274.26	1,274.26	1,274.26	1,274.26	1,274.26	E(HIP) = E(M-Before) × 50.50%
Health care Provider (HP) revenue	2,523.28	2,308.26	2,113.73	2,486.92	2,384.87	2,227.40	2,487.88	R(HP) = E(OOP) + E(HIP)
HP additional revenue	-	219.37	417.82	37.10	141.20	301.85	36.11	AR(HP) = R(HP)- E(M)
Rate of HP additional revenue	-	9.50%	19.77%	1.49%	5.92%	13.55%	1.45%	Rate[AR(HP)] = AR(HP)/R(HP)
Expected savings of medical expenditure	-	434.39	827.37	73.46	279.61	597.73	71.51	ESE(M) = E(M-Before)- E(M-After)
Expected savings of OOP expenditure	-	215.02	409.55	36.36	138.41	295.88	35.40	ESE(OOP) = E(OOP-Before)-E(OOP-After)
Rate of expected savings for medical expenditure	-	17.22%	32.79%	2.91%	11.08%	23.69%	2.83%	Rate[ESE(M)] = ESE(M)/E(M-Before)
Rate of expected savings for OOP expenditure	-	17.22%	32.79%	2.91%	11.08%	23.69%	2.83%	Rate[ESE(OOP)] = ESE(OOP)/E(OOP-Before)
	***Before* intervention value**	***After* intervention value -**R(I)** = 20%**			***After* intervention value -**R(I)** = 10%**			**Description** (**E:** Expenditure; **R:** Revenue; **AR:** Additional Revenue; **ESE:** Expected savings expenditure)
(Per TB patient by year)	Average	Average	Minimum	Maximum	Average	Minimum	Maximum	
Medical (M) expenditure	2,523.28	2,018.76	1,569.80	2,476.07	1,977.73	1,442.04	2,459.61	E(M)
Out-of-pocket (OOP) expenditure	1,249.02	999.29	777.05	1,225.65	978.98	713.81	1,217.51	E(OOP) = E(M) × 49.5%
Health Insurance Payments (HIP) expenditure	1,274.26	1,274.26	1,274.26	1,274.26	1,274.26	1,274.26	1,274.26	E(HIP) = E(M-Before) × 50.50%
Health care Provider (HP) revenue	2,523.28	2,273.54	2,051.31	2,499.91	2,253.23	1,988.06	2,491.76	R(HP) = E(OOP) + E(HIP)
HP additional revenue	-	254.78	481.51	23.84	275.50	546.03	32.16	AR(HP) = R(HP)- E(M)
Rate of HP additional revenue	-	11.21%	23.47%	0.95%	12.23%	27.47%	1.29%	Rate[AR(HP)] = AR(HP)/R(HP)
Expected savings of medical expenditure	-	504.52	953.48	47.21	545.55	1,081.24	63.67	ESE(M) = E(M-Before)- E(M-After)
Expected savings of OOP expenditure	-	249.74	471.97	23.37	270.05	535.22	31.52	ESE(OOP) = E(OOP-Before)-E(OOP-After)
Rate of expected savings for medical expenditure	-	19.99%	37.79%	1.87%	21.62%	42.85%	2.52%	Rate[ESE(M)] = ESE(M)/E(M-Before)
Rate of expected savings for OOP expenditure	-	19.99%	37.79%	1.87%	21.62%	42.85%	2.52%	Rate[ESE(OOP)] = ESE(OOP)/E(OOP-Before)

With the intervention, if R(I) = 30%, the total annual expenditure per TB patient is expected to decrease from $2523,28 to $2088.89, which is a decrease of $434.39 (17.22%), and the OOP expenditure is expected to decrease by $215.02 (17.22%). When R(I) = 50%, the total annual expenditure per TB patient is expected to decrease from $2523,28 to $2,243.67, which is a decrease of $279.61 (11.08%), and the OOP expenditure is expected to decrease by $138.41 (11.08%). If R(I) = 20%, the total annual expenditure per TB patient is expected to decrease from $2523,28 to $2018.76, which is a $504.52 (19.99%) decrease, and the OOP expenditure is expected to decrease by $249.74 (19.99%). When R(I) = 10%, the total annual expenditure per TB patient is expected to decrease from $2523,28 to $1,977.73, which is a decrease of $545.55 (21.62%), and the OOP expenditure is expected to decrease by $270.05 (21.62%).

Health care provider (HP) revenue is also mainly composed of the HIP and OOP. Based on the intervention design, the health insurance reimbursement rate of 50.50% (Table 3 by 2022) and HIP in 2022 will remain unchanged from the previous year (this payment rate will increase over time based on the negotiated rate). Therefore, the HIP is still $1,274.26 with the intervention. When R(I) = 30%, although the HP revenue decreased from $2523.28 to $2,308.26, the OOP decreased from $1,249.02 to $1,034.00. The HP will receive additional revenue of $219.37. The revenue/expenditure ratio increased from -6.09% to 9.50%. When R(I) = 50%, although the HP revenue decreased from $2523.28 to $2,384.87, the OOP decreased from $1,249.02 to $1,10.62. The HP will receive additional revenue of $141.20. The revenue/expenditure ratio increases from -6.09% to 5.92%. When R(I) = 20%, although the HP revenue decreased from $2523.28 to $2,273.54, the OOP decreased from $1,249.02 to $999.29. The HP will receive additional revenue of $254.78. The revenue/expenditure ratio increased from -6.09% to 11.21%. When R(I) = 10%, although the HP revenue decreased from $2523.28 to $2,253.23, the OOP decreased from $1,249.02 to $978.98. The HP will receive additional revenue of $275.50. The revenue/expenditure ratio increases from -6.09% to 12.23%.

## Discussion

BP has been used as an effective policy instrument to change health care providers’ practice behaviours, improve the quality of care, and control the cost of services. The BP intervention that was developed for this pilot study aims to achieve those goals through financial incentives to promote low-cost TB treatment (such as outpatient services instead of inpatient services), in order to reduce patients’ financial burdens, increase health care providers’ financial stability, and reduce the financial risk to health insurance funds while ensuring that TB treatment follows standard treatment guidelines in China. The estimated results from this simulation analysis showed that with a bounded payment intervention and a targeted hospitalization rate of 30%, patient OOP could be reduced by approximately 17.22%. Although the revenues of health care providers/institutions declined, their revenue/expenditure ratio increased from -6.09% to 9.50%, which made them financially more sustainable. Health insurance payments could be controlled accordingly with the negotiation payment rate over time. The simulation results showed that even with a higher hospitalization rate of 50% or a lower hospitalization rate of less than 30%, the potential benefits of the different stakeholders still hold more significance.

Based on data from the pilot area in 2022, 631 general TB patients received fully standardized treatment. The hospitalization rate for this population was 44.85%, and the average medical cost per patient was $1557.46. This result suggested that hospitalization rates can be significantly controlled (between 30 and 50% of our prediction) if patients follow standard TB treatment guidelines in China. The average medical cost for patients was close to the predicted standard cost for treatment guidelines. This result demonstrates this study’s effectiveness in implementing this BP model in the pilot area.

Although we have applied BPs to address TB patients’ health care service issues, this method applies to other similar chronic disease conditions to promote coordinated services, such as the balance between outpatient and inpatient care or daily based patient management and emergency services, throughout the entire treatment period to improve the efficiency and effectiveness of health care services. For example, for patients with diabetes, if provider payments can more effectively promote the control of blood glucose, diabetes could be prevented. This could subsequently lead to cost savings and health improvement over a longer period of time. Recently, payment intervention efforts in China have focused more on hospital services. BPs could be one of the policy tools that can help to achieve this objective.

The results of this study are based on simulations, which have many assumptions. The actual pilot experiments were performed in the selected pilot cities. The impact evaluation was planned to examine the actual effects of this innovative BP on the change in TB patients’ hospitalization rate, the improvement of health care services, the potential cost savings, and the potential financial impacts on the different stakeholders.

This study’s use of data from a specific pilot area, supported by the Gates Foundation as part of a BP initiative, brings several implications for the validity and robustness of its findings. The high relevance of the research is supported by the focus on tuberculosis patients within this pilot area, allowing for a precise examination of policy effects pre- and post-implementation. This setup offers a controlled environment, which facilitates the identification of causal relationships and enhances the study’s internal validity by minimizing external variable interference.

Additionally, the random selection of the pilot site helps mitigate sample bias, potentially making the findings more representative of a broader population of TB patients. However, there are inherent limitations to this approach. The data’s applicability to other regions may be limited due to social, cultural, and economic differences, which could impact the external validity of the findings. Specific environmental factors unique to the pilot site might also influence the outcomes, further challenging the robustness and general applicability of the results.

Looking ahead, future research aims to expand the study to multiple pilot areas to better generalize the results across different settings. This expansion intends to select cities with comparable social, cultural, and economic backgrounds to the original pilot area for control groups. Such an approach will likely reduce the impact of external variables and improve the internal validity of subsequent findings, thereby enhancing the overall reliability and applicability of the research in diverse environments.

## Conclusions

This study demonstrated the potential effectiveness of bundled provider payments as a policy tool for quality improvement and cost control in health care. The intervention that was developed for this pilot study, which promotes low-cost TB treatment through outpatient services, has shown promising results in reducing the financial burden on patients, increasing the financial stability of health care providers, and reducing the financial risk to health insurance funds.

Simulation analysis suggested that patient out-of-pocket costs could be reduced by approximately 17.22%, with a target hospitalization rate of 30%. Even with a greater hospitalization rate of 50% or a lower hospitalization rate of less than 30%, the benefits to stakeholders remain significant. Real-world data from the pilot area in 2022 support these findings, with hospitalization rates and average medical costs aligning closely with predictions.

This study faced several limitations that could influence the interpretation of the results. The simulation model adopted the China CDC’s recommended TB inpatient rate as the target hospitalization rate. We explored a range of plausible scenarios by simulating hospitalization rates of 30% and 50%, as well as 20% and 10%. Despite these varied simulations, we cannot discount the possibility that actual hospitalization rates might exceed our highest projections, potentially diminishing the perceived effectiveness of the interventions.

Furthermore, the baseline data utilized in this analysis were exclusively from 2020, a period heavily impacted by COVID-19 outbreaks. This might have altered both the number of patients and the types of services rendered, thereby affecting our simulation outcomes. Another potential limitation is the set of assumptions regarding the effectiveness of the intervention, which was predicated on an assumed initial hospitalization rate of 100%—a worst-case scenario.

Our financial forecasts relied on data from contagious disease hospitals in the pilot city, which might not accurately reflect the financial operations specific to TB due to the inclusion of other diseases in these data sets. Additionally, the treatment guidelines were used to define the benefit package for estimating payment rates. We hypothesized that providers would align their practices with these guidelines over time if properly incentivized, although this study did not capture any financial improvements linked to enhanced quality of healthcare services.

Cost considerations also extended to monitoring and evaluation efforts, traditionally seen as administrative costs. We did not anticipate these activities to necessitate additional funding beyond what has been historically noted. The study also did not address whether health care resources were used rationally or not; it merely set prices from a practical standpoint without assessing past issues of over- or under-treatment. Nonetheless, it is hoped that cost control measures will encourage providers to continuously address and mitigate resource overutilization.

Lastly, we assumed a static reimbursement rate for health insurance despite focusing on reimbursement levels as a key study area. Over time, we expect the reimbursement rate to increase gradually, alleviating the financial burden on individuals. Specifically, TB has been recognized as a chronic catastrophic illness, potentially leading to higher reimbursement rates for outpatient services under this designation. However, our simulations did not reflect these potential policy impacts, which should be investigated further based on pilot study results.

In light of the limitations identified in this study, several improvements can be envisioned for future research to refine the results further. Increasing the granularity of the target hospitalization rate to intervals of 5% or even 1% could provide more detailed insights into the impacts of varying hospitalization scenarios. Additionally, utilizing the most recent data would help mitigate the distortive effects of extraordinary events such as the COVID-19 pandemic, ensuring that outcomes reflect more typical service usage patterns.

To enhance the accuracy of financial analyses, it would be beneficial to use data that directly reflect the income and expenditures of TB patients, thereby avoiding the confounding effects of broader datasets that include other diseases. Moreover, employing more sophisticated methods to assess financial impacts related to improvements in quality of care would provide a deeper understanding of how healthcare practices affect economic outcomes.

Considering the dynamic nature of health insurance reimbursement rates, future studies could also explore the use of Markov models or similar analytical tools. These methods would allow researchers to more accurately simulate the long-term effects of changes in reimbursement policies on patients and healthcare providers alike.

In summary, this study offers valuable insights into the potential of BPs to enhance medical outcomes and control costs, particularly in managing costs associated with TB treatment. We validated the feasibility of the BP approach developed by the NHSA. This new payment model has a significant positive impact on improving the quality of medical services, alleviating the financial burden on patients, and enhancing the financial stability of health insurance funds, especially for TB patients. Additionally, BPs demonstrate their potential in the treatment of other diseases, especially chronic diseases, by promoting better coordination and continuity of medical services. These findings could provide valuable information for health care payment system reforms in other countries or regions throughout the world in the future.

## Data Availability

The data that support the findings of this study are available from the Bill & Melinda Gates Foundation, but restrictions apply to the availability of these data, which were used under license for the current study and are not publicly available. However, the data are available from the authors upon reasonable request and with the permission of the Bill & Melinda Gates Foundation.

## Abbreviations

AR: Additional Revenue
BP: Bundled Payment
CABG: Coronary Artery Bypass Grafting
CBP: Case-Based Payment
CDC: Centre For Disease Control
CMMI: Center for Medicare and Medicaid Innovation
COVID-19: Novel Coronavirus Pneumonia
CPIMS: Consumer Price Index For Medical Services
E: Expenditure
ESE: Expected Savings Expenditure
FFS: Fee-For-Service
HIP: Health Insurance Payments
HP: Health care Provider
ICD-9: International Classification of Diseases, Ninth Revision
ICD-10: International Classification of Diseases, Tenth Revision
NHSA: China National Health Security Administration
OOP: Out-of-pocket
QALYs: Quality-Adjusted Life-Years
R: Revenue
TB: Tuberculosis
UEBMI: Urban Employee Basic Medical Insurance
URBMI: Urban Resident Basic Medical Insurance
